# The Early Social Cognition Inventory (ESCI): An examination of its psychometric properties from birth to 47 months

**DOI:** 10.3758/s13428-021-01628-z

**Published:** 2021-09-10

**Authors:** Elena Hoicka, Burcu Soy-Telli, Eloise Prouten, George Leckie, William J. Browne, Erika Nurmsoo, Merideth Gattis

**Affiliations:** 1 School of Education, University of Bristol, Bristol, BS8 1JA UK; 2 Department of Psychology, University of Sheffield, Sheffield, UK; 3 Department of Psychology, Nevşehir Hacı Bektaş Veli University, Nevşehir, Turkey; 4 School of Psychology and Life Sciences, Canterbury Christ Church University, Canterbury, UK; 5 School of Psychology, Cardiff University, Cardiff, UK

**Keywords:** Social cognition, Theory of Mind, Survey development, Infants, Toddlers, Preschoolers

## Abstract

Social cognition refers to a broad range of cognitive processes and skills that allow individuals to interact with and understand others, including a variety of skills from infancy through preschool and beyond, e.g., joint attention, imitation, and belief understanding. However, no measures examine socio-cognitive development from birth through preschool. Current test batteries and parent-report measures focus either on infancy, or toddlerhood through preschool (and beyond). We report six studies in which we developed and tested a new 21-item parent-report measure of social cognition targeting 0–47 months: the Early Social Cognition Inventory (ESCI). Study 1 (*N* = 295) revealed the ESCI has excellent internal reliability, and a two-factor structure capturing social cognition and age. Study 2 (*N* = 605) also showed excellent internal reliability and confirmed the two-factor structure. Study 3 (*N* = 84) found a medium correlation between the ESCI and a researcher-administered social cognition task battery. Study 4 (*N* = 46) found strong 1-month test–retest reliability. Study 5 found longitudinal stability (6 months: *N* = 140; 12 months: *N* = 39), and inter-observer reliability between parents (*N* = 36) was good, and children’s scores increased significantly over 6 and 12 months. Study 6 showed the ESCI was internally reliable within countries (Australia, Canada, United Kingdom, United States, Trinidad and Tobago); parent ethnicity; parent education; and age groups from 4–39 months. ESCI scores positively correlated with household income (UK); children with siblings had higher scores; and Australian parents reported lower scores than American, British, and Canadian parents.

**Supplementary Information:**

The online version contains supplementary material available at 10.3758/s13428-021-01628-z.

Social cognition refers to a broad range of cognitive processes and skills that allow individuals to interact with and understand others (Gattis, 2018). Social cognition first emerges in infancy, and continues to develop through early childhood via the accumulation of different skills. Nonetheless, many studies focus on only one socio-cognitive skill within a narrow age range (Baron-Cohen et al., 1985; Behne et al., 2012; Carpenter, Akhtar, et al., 1998a; Denham, 1986; Repacholi & Gopnik, 1997). Those studies that do look at multiple socio-cognitive skills across a wider age range demonstrate that this approach is a time-consuming and expensive activity involving multiple lab visits and a battery of tasks (Carpenter, Nagell, et al., 1998b; Hilbrink et al., 2013; Sakkalou et al., 2013). The goal of the current studies was to develop a short parent-report measure of social cognition from birth through to 47 months, the Early Social Cognition Inventory (ESCI), and evaluate the convergent validity and reliability of the measure. The ESCI would allow researchers to efficiently measure socio-cognitive development, including: (1) a comparison of social cognition to other skills and abilities, both cross-sectionally and longitudinally, with a wide age range; and (2) a control for social cognition experiments, covering a wide age range.

The first socio-cognitive skill to emerge developmentally can also be seen as a gateway to social cognition more generally: orienting to social partners. New-born infants attend to faces more than other visual stimuli and within a few months, recognize familiar faces (Farroni et al., 2005; Johnson et al., 1991). Faces are valuable dynamic stimuli, providing cues not only about identity but also about the attentional focus and emotional state of social partners (Frith, 2008). Across the first year, infants increasingly orient to objects as well as faces, eventually shifting attention back and forth between an object and person, a process known as joint attention (Morales et al., 2000; Perra & Gattis, 2010, 2012). Around 1 year of age, most children are capable of gaze- and point-following, and in addition, produce points to communicate with others (Brooks & Meltzoff, 2005; Camaioni et al., 2004; Carpenter, Nagell, et al., 1998b; Liszkowski et al., 2004). During approximately the same developmental period as the emergence of joint attention, children begin to copy the actions of others in two different ways: mimicry (copying actions without necessarily understanding the intentions behind them) and imitation (copying actions while understanding the intentions behind them) (Behne et al., 2012; Carpenter, Akhtar, et al., 1998a; Carpenter, Nagell, et al., 1998b; Gergely et al., 2002; Liszkowski, 2005; Liszkowski et al., 2004; Liszkowski et al., 2006; Liszkowski et al., 2007; Sakkalou & Gattis, 2012; Tomasello, 1995). Longitudinal evidence from researcher-administered tests indicates that joint attention and at least some forms of copying the actions of others are linked. For instance, Carpenter, Nagell, et al. (1998b) used Guttman analysis to demonstrate that joint attention, gaze and point following, children’s own pointing, and imitation were developmentally related.

Social cognition also refers to knowledge and understanding of social partners, including their desires, emotions, and beliefs (Barna & Legerstee, 2005; Denham, 1986; Wellman & Woolley, 1990). By 18 months, most children understand others’ desires (Repacholi & Gopnik, 1997), and by around 2 years, they understand others’ emotions and perspectives (Denham, 1986; Moll & Tomasello, 2006). All of these skills involve taking another’s point of view (Denham, 1986; Gopnik & Slaughter, 1991; Moll & Tomasello, 2006). Finally, by around 4.5 years, children show explicit false belief understanding (Wellman et al., 2001), though implicit false belief understanding may be evident as young as 17 months (Onishi & Baillargeon, 2005; Southgate et al., 2010; Southgate et al., 2007). Studies with American and Australian 3–5-year-olds showed that children generally pass a series of social cognition tasks in the same order, first understanding desires, then beliefs, knowledge, false beliefs, and complex emotions (Shahaeian et al., 2011; Wellman & Liu, 2004). However, this order is slightly different in countries such as China, Iran and Turkey where children tend to acquire knowledge before beliefs (Selcuk et al., 2018; Shahaeian et al., 2011; Wellman et al., 2006).

Longitudinal evidence helps developmental scientists more accurately identify the ages at which specific socio-cognitive skills emerge, as well as the order of emergence across skills (e.g., Carpenter, Nagell, et al., 1998b; Wellman & Liu, 2004). Longitudinal evidence is also essential for evaluating the continuity (consistency of the group across time) and stability (consistency of individual rank across time) of socio-cognitive skills (Bornstein et al., 2017). As a result, longitudinal evidence plays an important role in describing developmental trajectories, assessing individual performance, and in evaluating theoretical questions about relations between different skills and processes. At present, most longitudinal studies of social cognition have relied on lab-based researcher testing (Carpenter, Nagell, et al., 1998b; Wellman & Liu, 2004), which is time-consuming and expensive (e.g., seven visits per child, Carpenter, Nagell, et al., 1998b). Longitudinal studies of social cognition have also tended to cover a restricted age range, perhaps because of the time and related costs involved.

A potentially more efficient approach to measuring social cognition more broadly is to use parent-report measures. The Children’s Social Understanding Scale (CSUS) (Tahiroglu et al., 2014) achieves this task with a 42-item survey for children with typical development from 2.5 to 7 years, asking questions covering children’s understanding of beliefs, knowledge, perception, desires, intentions, and emotions, which showed good internal reliability, and correlated well with researcher-administered social cognition tasks. The Theory of Mind Inventory (ToMI) (Hutchins et al., 2012) was designed to assess Theory of Mind development in children from 2 to 18 years, and shows good internal reliability across questions including perspective-taking, others’ thoughts and emotions, joint attention, false belief, and the appearance–reality distinction. Importantly however, the lower age limit of these surveys is 2 years, despite the fact that social cognition is already developing in the first year. Research therefore needs to determine whether socio-cognitive skills can be measured reliably by parents in children from the first year, when these skills first emerge. Our survey will determine whether 0–47-month-olds’ socio-cognitive development can be measured with one survey. The questions we designed were based on past lab experiments, including attention to faces, joint attention, gaze and point following, pointing, mimicry, imitation, and understanding intentions, mistakes, desires, perspectives, emotions, the appearance reality distinction, beliefs, and knowledge (see Study 1 for details on survey construction) (Baron-Cohen et al., 1985; Behne et al., 2012; Carpenter, Akhtar, et al., 1998a; Carpenter, Nagell, et al., 1998b; Denham et al., 2002; Farroni et al., 2005; Frith, 2008; Gergely et al., 2002; Johnson et al., 1991; Jones, 2007; Liszkowski, 2005; Liszkowski et al., 2004; Liszkowski et al., 2006; Moll & Tomasello, 2006; Repacholi & Gopnik, 1997; Sakkalou & Gattis, 2012; Tomasello, 1995; Wellman et al., 2001). The current project also involved comparing parent-report measures to a subset of analogous researcher-administered tasks to determine the parent report measures’ concurrent validity.

One use for a short parent-report measure of social cognition is to easily determine how social cognition relates to a variety of other areas of development, across a wide age range, either cross-sectionally or longitudinally. We already know that social cognition influences other aspects of development in typically developing children. The ability to engage in joint attention in infancy predicts executive function later on, while the amount of joint attention infants engage in predicts vocabulary later on (Miller & Marcovitch, 2015; Morales et al., 2000). The ability to follow gaze and point, point, and direct gaze in infancy predict receptive and expressive language (Laakso et al., 1999; Moberg et al., 2017). The ability to imitate in infancy also predicts expressive language (Laakso et al., 1999), and is linked to extraversion (Hilbrink et al., 2013). Preschoolers’ ability to understand emotions negatively predicts how hostile children will become later on (Choe et al., 2013). Finally, preschoolers’ Theory of Mind, or ability to understand false beliefs, predicts how well children will be liked in the future, and how hostile children will become (Choe et al., 2013; Slaughter et al., 2002). Given that many components of social cognition predict a variety of skills in children, future research would benefit from a short, easy to use tool to examine these and further relationships. Other surveys have been used extensively in this manner. For instance, Tsao et al. (2004) found that speech discrimination determined via an experiment at 6 months predicted language development, measured by the MacArthur-Bates Communicative Development Inventory, at 2 years. Similarly, Libertus and Needham (2014) found that 3-month-olds’ face preference determined via an experiment correlated with their motor activity, via the Infant Behavior Questionnaire.

Another use for a short parent-report measure of social cognition is to act as a baseline measure in experiments where socio-cognitive skills are a dependent variable. For instance, some between-subjects tasks examined whether children imitated intentional actions, but not accidental or irrelevant actions (Carpenter, Akhtar, et al., 1998a; Gergely et al., 2002; Sakkalou & Gattis, 2012). However, where results are positive, there is always a chance that the experimental group had more advanced socio-cognitive development to begin with. Using a short parent-report measure of social cognition as a baseline could control for variation between groups, reducing this potential problem.

Finally, the ESCI could be a valuable tool in practice. For instance, medical professionals, such as doctors and health visitors, might be able to use it as a screening tool to identify children who are not following typical developmental trajectories. Early years educators and parents could also use the ESCI to determine how advanced children’s social cognition is in order to pitch communication and activities at the right level.

The current study sought to create a short parent-report measure of social cognition from birth to 47 months. The study included constructing the ESCI (*N* = 295, Study 1) and validating the ESCI with a separate sample, (*N* = 605, Study 2). We also sought convergent validity by comparing parent reports to a battery of researcher-administered social cognition tasks to ensure that parent reports related to more objective, frequently used researcher-administered measures on another separate sample, (*N* = 84, Study 3). We measured test–retest reliability at 1 month (*N* = 46, Study 4), as well as longitudinal stability at 6- (*N* = 140) and 12-month (*N* = 39) intervals and examined inter-rater reliability between parents (*N* = 36, Study 5, based on a subset of participants from Studies 1–4). All data (Studies 1–4) were also compiled to examine internal reliability within different demographic groups (different countries; levels of education; parent ethnicity; children mono- or multilingual); how items and the scale change across age; and to examine demographic differences (child gender, siblings, childcare hours, child mono- or multilingual, parent gender, parent age, parent education, household income; Study 6).

## Study 1: Survey construction

The goal of developing the ESCI was to design an inventory that could (1) be used across a wide age range (birth through 47 months), and (2) identify the emergence of socio-cognitive skills that, once achieved, would remain. The latter was important as the ESCI was intended to capture the developmental progression of socio-cognitive skills. Therefore, socio-cognitive behaviors that emerge temporarily, such as stranger anxiety, should not be included.

The first author conducted a literature review of diverse socio-cognitive skills across the 0 to 47-month age range. A general search for terms like “social cognition” or “Theory of Mind” alongside terms such as “preschool*”; “toddler*” and “infan*” was not a good strategy, as one such search yielded over 90,000 results on PsycInfo. Therefore, the search instead focussed on review articles, and research articles that looked at a range of socio-cognitive skills across a wide age range which overlapped with our target age range. Two instruments evaluating socio-cognitive skills in children 2 years and older, the Perceptions of Children’s Theory of Mind Measure—Experimental Version (PCToMM-E) (Hutchins, et al., 2012), and the CSUS (Tahiroglu, et al., 2014), suggested several socio-cognitive skills to tap into, including, emotion, intention, desire, perception, belief, and knowledge. Indeed, these surveys showed good reliability, suggesting these skills are related.

However, we also need to cover socio-cognitive skills which develop before two years. We began with theoretical and review papers to examine what socio-cognitive skills are present in children under 2 years, and also examined empirical work that covered a range of socio-cognitive skills and ages under 2 years. Pedagogy Theory has been proposed by Csibra and Gergely (2006), suggesting socio-cognitive skills emerging from birth support knowledge transfer in humans. These socio-cognitive skills include face preference in new-borns, gaze following, goals, pointing, and imitation. Empirical research also lends supports to the idea that several socio-cognitive skills develop in the first years. A longitudinal study by Carpenter, Nagell, et al., (1998b) from 9–15 months measured joint attentional engagement, gaze and point following, imitation of actions on objects (tapping into intention understanding), as well as imperative and declarative (point) gestures. This study found these skills are related, emerging in a consistent order across children. While most surveys and experiments focus on children’s understanding of others’ social cognition, Meltzoff (2007) suggested the “like-me” hypothesis, that children come to understand others’ socio-cognitive processes by comparing them to their own. This paper provides a theory of how social cognition emerges in infancy, and includes skills and concepts such as perception, emotion, imitation, gaze-following, and goals. Therefore, we chose to include items which considered whether children understand their own, as well as others’, social cognition.

After generating a list of socio-cognitive skills, we next generated items that linked socio-cognitive skills to experimental tasks that captured these skills. For instance, in the Carpenter, Nagell, et al., (1998b) study, an experimenter held one item in each hand, and looked back and forth between the child and the item, to determine if the child would gaze follow toward the item. This led to the question, “Does your child follow where you look in order to look at the same thing as you?” Other items were created in the same way (see Table 1 for experimental sources for items). For items focussing on the child’s understanding of their own social cognition, we adapted some of the items derived from experiments’ focussing on others’ social cognition to instead focus on the child. For instance, the item, “Does your child understand what it means for others to make mistakes? E.g., that they dropped a plate by accident.” was based on an experiment by Carpenter, Akhtar, et al (1998a) which children had to distinguish an intentional action from a mistake. We then adapted this item to focus on the child’s understanding of their own mistakes, “Is your child aware of his/her own mistakes? E.g., if s/he drops something by accident.” This process led us to create 23 items that involved skills that experimental research found emerged from birth (e.g., face preference) (Farroni et al., 2005; Johnson et al., 1991), to just beyond the 47-month mark (false belief understanding) (Baron-Cohen et al., 1985). The next step was to test the items with an initial pool of participants (DeVellis, 2017).

**Table 1:** ESCI items

Item	Question	Skill	*r*	F1	F2	25% pass	50% pass	75% pass	Source	Age
9	Does your child look back and forth between you and an object, instead of only looking at you or only at an object?	Joint attention	.45*	**0.60** (0.39)	0.36 (**0.41**)	0–1	4–5	8–9	(Bakeman & Adamson, 1984; Carpenter, Nagell, et al., 1998b)	6
1	Does your child follow where you look in order to look at the same thing as you?	Gaze-following	.52*	**0.74** (**0.72**)	0.36 (– 0.18)	2–3	4–5	8–9	(Brooks & Meltzoff, 2005; Carpenter, Nagell, et al., 1998; Morales et al., 2000; Perra & Gattis, 2010)	3
8	Does your child follow where you point to look at the same things as you?	Point-following	.64*	**0.87** (**0.82**)	0.36 (0.41)	4–5	6–7	8–9	(Carpenter, Nagell, et al., 1998)	11
11	Does your child copy others in order to achieve the same goal? E.g., copying pressing a button to make a song play on a toy.	Imitation	.64*	**0.88** (**0.85**)	0.41 (0.48)	4–5	6–7	8–9	(Carpenter, Akhtar, et al., 1998; Carpenter, Nagell, et al., 1998; Gergely et al., 2002; Hilbrink et al., 2013; Sakkalou et al., 2013; Sakkalou & Gattis, 2012)	12
7	Does your child perform actions intentionally? E.g., stack blocks on purpose, instead of by trial and error.	Own intentions	.71*	**0.89** (**0.86**)	0.24 (0.33)	6–7	8–9	12–13	(Carpenter, Nagell, et al., 1998)	9
3	Is your child aware of their own desires? E.g., prefer chocolate over broccoli.	Own desires	.70*	**0.90** (**0.87**)	0.12 (0.21)	6–7	8–9	12–13	(Repacholi &Gopnik, 1997; Wellman & Liu, 2004)	18
14	Does your child point to get something from you? E.g., to get a toy that is out of reach.	Imperative pointing	.77*	**0.91** (**0.78**)	0.11 (0.49)	8–9	10–11	12–13	(Camaioni et al., 2004; Kovács et al., 2014; McGillion et al., 2017)	12
17	Is your child aware of other people’s emotions? E.g., happy, sad, angry, etc.	Others’ emotions	.70*	**0.84** (**0.88**)	– 0.13 (– 0.05)	6–7	12–13	20–21	(Barna & Legerstee, 2005; Denham, 1986)	9/30
16	Does your child point to share information with you? E.g., point to show you a dog in the park.	Declarative pointing	.79*	**0.92** (**0.86**)	0.07 (0.33)	10–11	12–13	14–15	(Camaioni et al., 2004; Liszkowski, 2005; Liszkowski et al., 2004; Liszkowski et al., 2006; Liszkowski et al., 2007; McGillion et al., 2017)	12
6	Is your child aware of his/her own mistakes? E.g., if s/he drops something by accident.	Own mistakes	.80*	**0.95** (**0.90**)	0.11 (0.11)	10–11	12–13	16–17	(Carpenter, Akhtar, et al., 1998; Sakkalou & Gattis, 2012)	14
4	Is your child aware that other people may know the same information they do? E.g., they know where a certain book is kept, and they know their dad knows where that book is kept too.	Others’ knowledge same	.78*	**0.92** (**0.87**)	– 0.01 (– 0.02)	12–13	16–17	22–23	(Baron-Cohen et al., 1985; Onishi & Baillargeon, 2005; Southgate et al., 2010; Southgate et al., 2007; Wellman & Woolley, 1990)	33
13	Is your child aware of their own emotions? E.g., happy, sad, angry, etc.	Own emotions	.69*	**0.81** (**0.89**)	– 0.10 (– 0.17)	12–13	18–19	24–25	(Barna & Legerstee, 2005; Denham, 1986)	9/30
20	Does your child understand what it means for others to make mistakes? E.g., that they dropped a plate by accident.	Others’ mistakes	.80*	**0.93** (**0.89**)	– 0.12 (– 0.13)	16–17	18–19	22–23	(Carpenter, Akhtar, et al., 1998; Sakkalou & Gattis, 2012)	14
21	Does your child perform actions with specific goals in mind? E.g., stacking blocks specifically to make a house.	Own goals	.75*	**0.87** (**0.82**)	– 0.04 (– 0.25)	14–15	20–21	26–27	(Carpenter et al., 2005; Southgate et al., 2009)	12
2	Is your child aware of other people’s motives? E.g., that they might give someone a gift in order to make them happy.	Others’ motives	.71*	**0.84** (**0.77**)	– 0.08 (– 0.18)	14–15	20–21	32–33	(Curenton, 2011)	43
19	Is your child aware that sometimes other people don’t know the same information they do? E.g., child might know where a toy is, but dad might not.	Others lack knowledge	.70*	**0.88** (**0.80**)	– 0.18 (– 0.32)	20–21	26–27	36–37	(Baron-Cohen et al., 1985; Onishi & Baillargeon, 2005; Southgate et al., 2010; Southgate et al., 2007; Wellman & Bartsch, 1988; Wellman & Woolley, 1990)	17/38
10	Does your child understand that sometimes things aren’t as they appear? E.g., something that looks hard might feel soft.	Appearance–reality	.70*	**0.84** (**0.77**)	– 0.12 (– 0.21)	18–19	28–29	40–41	(Flavell et al., 1983; Gauvain & Greene, 1994)	32
15	Does your child understand that sometimes other people have different desires to themselves? E.g., other people might like broccoli, even if they don’t.	Others’ desires different	.70*	**0.89** (**0.83**)	– 0.33 (– 0.35)	22–23	28–29	32–33	(Repacholi & Gopnik, 1997; Wellman & Liu, 2004)	18
18	Is your child aware that other people may have the same beliefs as them? E.g., that dogs are the best animals.	Others’ beliefs same	.68*	**0.88** (**0.83**)	– 0.36 (– 0.40)	22–23	28–29	36–37	(Flavell et al., 1990; Wellman & Liu, 2004)	37
5	Is your child aware of other people's perspectives? E.g., could they tell sometimes they can see something, but someone else can’t, because it’s not in their line of sight.	Others’ visual perspectives different	.64*	**0.82** (**0.70**)	– 0.11 (– 0.33)	20–21	30–31	*NA*	(Moll & Tomasello, 2006)	24
12	Is your child aware that sometimes other people don’t have the same beliefs as them? E.g., your child might think dogs are the best animal, but they understand that their sister thinks cats are the best animal.	Others’ beliefs different	.58*	**0.83** (**0.77**)	– 0.47 (– 0.21)	30–31	34–35	40–41	(Flavell et al., 1990; Wellman & Liu, 2004)	37

*Note* Spearman’s Rho correlations between the final items and total summed scale (r); and factor loadings for the exploratory factor analysis (Study 1), and a second exploratory factor analysis (Study 2, in brackets). Numbers are in bold for the factor for which the item loaded best. Construct refers to the target construct the item evaluated. 25%; 50%; 75% pass refers to the age (in months) by which we would expect 25%; 50%; 75% of children to pass each item based on all 4 samples combined (see Appendix A). Source indicates the research the items are based on. Age is the earliest age at which children were previously observed to have each skill. Where there are two ages, the younger age was determined with an implicit measure (e.g., eye-tracking), while the older age was determined with an explicit measured (e.g., verbal response). *p< .001; F1= Factor 1, F2 = Factor 2.

## Method

### Participants

Participants were tested on a preliminary version of the survey. There have been several methods suggested for determining sample size for survey construction, including ten participants per item (Tabachnick & Fidell, 2007), which would lead to 230 participants for our original 23-item survey. Therefore, to be conservative we aimed for over 250 participants to account for participants who may need to be cut, e.g., if they were too young, or reported their age wrong. We obtained surveys for 295 children. Participants were recruited online through Facebook advertising across countries worldwide for which English was the official language, press releases, Bounty packs within Sheffield, United Kingdom, and social media. Adverts were targeted at adults over 18 years who had a child from birth to 3 years. All participants completed a demographics survey (see Table 2). We do not report household incomes of samples that had fewer than five participants in a country. Ethical approval was obtained from the Psychology Department at the University of Sheffield for the projects, “Using parent reports to learn about early humour, pretending, deception, creativity, social cognition, actions, and language”, Reference Number 003095, and, "The relationship between humour development and social cognition from 3 months to 47 months: A lab study", Reference Number 013845. Parents who completed the survey on babylovesscience.com ticked boxes online to indicate their consent for the survey. Parents who completed the survey in the lab ticked boxes and signed a paper consent form. There was no reward for participation, unless participants repeated the survey 6 or 12 months later, or the child’s other parent also completed the survey (see Study 5).

**Table 2:** Participant Information

*N*	Study 1	Study 2	Study 3	Study 4
	295	605	84	63
**Children’s Age:**				
Mean (months; days)	17;12	25;20	23;19	32;1
Range	0;17 – 47; 10	0;17 – 47;24	3;7 – 46;5	7;22-47;25
SD	11;29	11;21	13;20	11;26
**Children’s Gender:**				
Female	140	298	40	25
Male	154	307	44	38
Not reported	1	0	0	0
**Children’s Ethnicity:**				
Black	2	14	0	0
East Asian	4	3	0	0
Hispanic	2	1	0	0
Pacific Islander	0	1	0	0
South Asian	5	10	0	0
White	249	515	79	61
Of Mixed Ethnicity	7	18	4	2
Other (not specified)	21	32	0	0
Not reported	4	11	1	0
**Country:**				
Australia	123	10	0	0
Canada	10	15	0	0
Trinidad and Tobago	0	16	0	0
United Kingdom	103	436	84	63
United States of America	27	76	0	0
Other Country	29	43	0	0
Not reported	3	9	0	0
**Child’s Language**				
English only	229	463	64	32
English and another language(s)	58	101	13	30
Other language only (monolingual)	1	3	0	0
Other languages only (multilingual)	0	2	0	0
English, another language unclear	0	24	7	0
Not reported	7	12	0	1
**Siblings**				
Yes	102	258	41	45
No	187	315	36	18
Not reported	6	32	7	0
**Childcare hours**				
Mean	*NA*	17.15	12.46	16.07
Range	*NA*	0-75	0-40	0-47.5
SD	*NA*	15.01	12.06	13.11
Not reported	295	165	6	0
**Parents’ Age**				
Mean (years)	32.12	33.47	34.20	35.32
Range	18 – 48	18 – 46	22 – 43	27-44
SD	5.26	4.89	3.79	4.20
Not reported	2	33	7	0
**Parents’ Gender**				
Female	288	536	72	62
Male	5	36	2	1
Not reported	2	33	6	0
**Parents’ Ethnicity:**				
Black	3	17	1	0
East Asian	6	8	0	0
Hispanic	1	2	0	0
South Asian	7	9	0	0
White	263	500	75	63
Of Mixed Ethnicity	3	8	1	0
Other (not specified)	7	27	0	0
Not reported	4	34	7	0
**Parents’ Education**				
High school	37	60	15	6
Community College	31	33	0	0
Undergraduate Degree	111	210	36	30
Postgraduate Degree	111	289	33	27
Not reported	5	13	0	0
**Household Income**				
Australia: N	60	6	*NA*	*NA*
Mean	$123,250 AUD	$112,500		
Range	$30,000 – $350,000	$60,000-$200,000		
SD	$63,557	$61,298		
Canada: N	6	11	*NA*	*NA*
Mean	$115,000 CAD	$111,636		
Range	$60,000 – $200,000	$13,000 – $200,000		
SD	$52,154	$59,333		
Trinidad and Tobago: N	*NA*	8	*NA*	*NA*
Mean		$367,625 TTD		
Range		$130,000 – $630,000		
SD		$212,057		
United Kingdom: N	65	260	74	58
Mean	£58,754 GBP	£62,075	£53,980	£65,414
Range	£10,000 – £155,000	£6,000 – £750,000	£9,000 – £120,000	£24,000-£160,000
SD	£28,727	£52,855	£22,037	£27,339
United States of America:				
N	21	64	*NA*	*NA*
Mean	$82,190 USD	$132,563		
Range	$15,000 – $200,000	$20,000 – $250,000		
SD	$48,936	$61,275		
**Recruited**				
babylovesscience.com	295	552	0	0
University of Sheffield Cognitive Development Lab	0	53	84	0
Cardiff University’s Centre for Human Developmental Science	0	0	0	63

### Measures

#### Early Social Cognition Inventory (ESCI)

Participants completed the ESCI on www.babylovesscience.com using their own computer. The initial survey consisted of 23 items (see Table 1 for the final 21 items, after one item was dropped since it did not increase with age, and another item was dropped as it loaded more strongly with the age factor than the social cognition factor in the exploratory factor analysis, as discussed in the Results section). Examples of questions included, “Does your child follow where you look to look at the same things as you?” and, “Is your child aware of their own emotions?” Participants were required to respond either yes/no to each question, or could leave the item blank if the answer was “no” to save time. Each “Yes” response was summed to give a final score of 0–21 out of 21.

## Results

None of the ESCI items (*N* = 295) were collinear (all *Spearman’s Rho, r* < .860). We next ran binary logistic regression with each item as the dependent variable, and age in months as the independent variable to examine whether the percentage of positive responses to each item generally increased with age, or whether any items were transient phases. All items positively correlated with age (*N* = 295, *β* > .034, *Wald >* 10.53, *p* < .002), except item 22, “Does your child like to look at faces?” which correlated negatively with age (*N* = 295, *β* = – .119, *Wald* = 5.58, *p* = .018). We therefore cut this item as we deemed it not useful for tracking the increasing development of social cognition from birth to 47 months.

After removing item 22, we checked whether children as young as 0 months showed variation in scores. The mean summed score of the 22 items at 0 months (*N* = 4), was 1.00 (*SD* = 1.41, *range* = 0–3), suggesting the ESCI shows variation from birth, so we kept children as young as 0 months. We next examined whether each item correlated with the total ESCI score (the total number of “yes” responses across the remaining 22 items) using *Spearman’s Rho, r* > .3, *p* < .05 (Pedhazur & Schmelkin, 1991). All items correlated with the total ESCI score (all *r* > .32, *p* < .001). Internal reliability for the remaining 22 items was excellent, *Kuder–Richardson Formula 20* (*KR20*) = 0.94.

We then performed an exploratory factor analysis for binary items in R (Starkweather, 2014) using the psych package (Revelle, 2014). When looking at the scree plot two factors load at eigenvalues above 2, while a third factor was very close to 1, and all other factors were below 1. Parallel analysis suggested only two of these factors should be retained. We therefore ran a factor analysis for binary items with two factors. However, we found that all items loaded best onto Factor 1, at a value of .30 or greater, except item 23, “Does your child copy others for no clear reason? E.g., raises arm because someone else did, with no clear goal (other than to raise one ' s arms).” which loaded best onto Factor 2. Therefore, we re-ran the analysis without item 23.

**Fig. 1:**
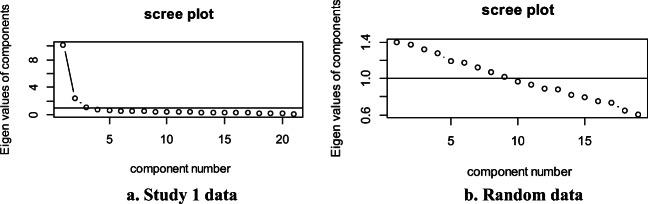
Parallel analysis for Study 1

When looking at the scree plot, again, two factors load at eigenvalues above 2, while a third factor was very close to 1, and all other factors were below 1. Parallel analysis again suggested only two of these factors should be retained (see Fig. 1). We therefore ran a factor analysis for binary items with two factors. This accounted for 80% of the variance. Table 1 shows the factor loadings for each item. All items loaded onto Factor 1 at a weighting of .44 or more, which accounted for 71% of the variance of the model. This factor appears to capture social cognition more generally. Seven items loaded onto Factor 2 at a weighting of .30 or more; or – .36 or less, which accounted for 9% of the variance of the model. Overall, items that loaded more strongly in a positive direction on Factor 2 were those that were passed at an earlier age (before 5 months, see Table 1). Items that loaded more strongly in a negative direction were those that were passed at a later age (from 27 months, see Table 1). Therefore, the two-factor structure picked up on social cognition overall, and age, which we aimed to capture in the ESCI. However, no distinct conceptual categories, such as intentions or pointing, nor own versus others’ social cognition, were captured by the factor structure. While some items loaded onto both factors, we put in bold the factor that each item loaded onto best (see Table 1).

See Table 3 for the final descriptive statistics, by year, for the 21-item ESCI. We then re-checked whether children as young as 0 months showed variation in scores, which did not change as none of the children passed item 23. We next re-examined whether each item correlated with the total ESCI score (the total number of “yes” responses across the remaining 21 items) using *Spearman’s Rho*. All items correlated with the total ESCI score (all *r* > .44, *p* < .001, see Table 1). Internal reliability for the remaining 21 items was excellent, *KR20* = 0.95.

**Table 3:** Descriptive statistics for age and ESCI scores by year in each study, and total scores for the researcher-administered social cognition tasks in Study 3

	0	1	2	3
**Study 1** *N* Age *M* *Range* *SD* ESCI *M* *Range* *SD*	125 6;9 0;17–11;25 3;6 4.74 0–16 3.73	86 18;0 12;2–23;21 3;7 13.02 3–21 3.55	54 29;7 24;0–35;10 3;10 16.96 10–21 2.93	30 40;21 36;21–47;10 2;26 19.27 10–21 2.68
**Study 2** *N* Age *M* *Range* *SD* ESCI *M* *Range* *SD*	61 7;7 0;17–11;27 3;17 4.66 0–16 4.09	217 17;12 12;0–23;27 3;12 11.50 0–20 3.98	195 29;18 24;0–35;22 3;12 16.01 0–21 4.39	132 42;4 36;4–47;24 3;17 19.05 12–21 2.07
**Study 3** *N* Age *M* *Range* *SD* ESCI *M* *Range* *SD* Tasks *M* *Range* *SD*	23 7;29 3;7–11.47 2;14 5.35 0–13 3.56 1.65 0–6 1.82	23 16;29 13;15–22;17 3;2 11.26 7–17 2.70 3.48 1–7 1.83	16 30;15 24;24–34;27 3;2 16.69 9–21 2.94 5.19 2–8 1.97	22 41;29 36;10–46;6 3;0 17.86 13–21 2.77 6.68 2–10 2.19
**Study 4** *N* Age *M* *Range* *SD* ESCI *M* *Range* *SD*	4 9;10 7;22–11;9 1;14 6.00 1–9 3.56	14 18;17 13;9–23;8 3;10 12.71 8–18 2.95	15 29;17 24;10–35;20 3;23 17.40 12–21 2.59	30 42;17 36;5–47;25 3;15 19.57 15–21 1.43

## Discussion

Study 1 found that 21 of the 23 ESCI items formed a cohesive scale, in which all items correlated with the total score; the items showed good internal reliability; and an exploratory factor analysis showed that the items formed a cohesive structure. Study 2 examined whether we could replicate internal reliability and the factor structure in a separate sample of participants.

## Study 2: Replication

### Method

#### Participants

Based on research suggesting ten participants per item are needed to construct surveys (Tabachnick & Fidell, 2007), we would need 210 participants for our final 21-item survey. However, as our goal in Study 6 was to examine demographic differences with small effect sizes, we would need a total of 787 children for a two-tailed small correlation (*f* = 0.1) with *α* = 0.05, power = 0.8; for regression analyses including linear regression, ANOVA, and ANCOVA (Faul et al., 2007). Therefore, to be conservative we aimed for 600 participants in Study 2, allowing for samples from Studies 1 and 2 to reach 787, accounting for attrition. We obtained surveys for 605 children in Study 2. Participants were recruited as in Study 1. All participants completed a demographics survey (see Table 2). There was no reward for participation, unless participants repeated the survey 6 or 12 months later, or the child’s other parent also completed the survey (see Study 5).

#### Measures

Parents completed the final 21-item ESCI as described in Study 1 on their own computer through www.babylovesscience.com; or through Qualtrics via a laptop in the lab while their child participated in an unrelated study.

### Results

See Table 3 for the descriptive statistics for children’s ages and ESCI scores, by year. Internal reliability for Study 2 (*N* = 605) on the 21 items of the ESCI was excellent, *KR20* = 0.93. This suggests that the 21 items form a coherent scale to capture early social cognition. We then performed an exploratory factor analysis for binary items in R (Starkweather, 2014) using the psych package (Revelle, 2014) with a two-factor structure, as in Study 1. This accounted for 76% of the variance. Table 1 shows the factor loadings for each item (in brackets). All 21 items loaded onto Factor 1 at a weighting of .39 or more, which accounted for 66% of the variance of the model. Seven items loaded positively onto Factor 2 at a weighting of .33 or more, while five items loaded negatively on Factor 2 at a weighting of – 0.32 or less, which accounted for 10% of the variance of the model. Five of the seven items which had a factor loading greater than 0.30 or lower than – 0.30 in Study 1 were again captured in Study 2 for Factor 2, however Study 2 captured more items overall, perhaps due to the larger sample size. Compared to Study 1, again, Factor 1 represented social cognition more generally, while Factor 2 represented age, with items loading more positively onto Factor 2 being passed at younger ages; and items loading more negatively onto Factor 2 being passed at older ages.

### Discussion

Study 2 replicated the internal reliability and factor structure found in Study 1. Therefore, the ESCI appears to consistently work. However, we also wanted to determine whether the ESCI correlated with an external social cognition measure. Therefore, in Study 3, we compared a separate sample of children’s scores on the ESCI to their performance on a battery of researcher-administered social cognition tasks.

## Study 3: Convergent validity

### Method

#### Participants

A power analysis found 84 children were needed to detect a two-tailed medium correlation (*r* = 0.3) (Tahiroglu et al., 2014) with *α* = 0.05, power = 0.8 (Faul et al., 2007). Participants were recruited through Bounty packs within Sheffield, United Kingdom, press releases, and Facebook advertising within Sheffield, United Kingdom; and their demographic details can be found in Table 2. This sample was selective as additional children were not included because children did not want to participate (e.g., stating they did not want to play the game, or e.g., crying for younger children; *N* = 26; 16 male, 10 female; *M* age = 22;14; *SD* = 10;1). Additional children were not included due to technical problems with the videos (*N* = 3), or experimental error (*N* = 2). All participants completed a demographics survey (see Table 2). Children received a book for participating.

#### Measures

##### ESCI

Parents completed the final 21-item ESCI through Qualtrics on a laptop in the lab before their child participated in the social cognition tasks.

##### Social cognition tasks

Children participated in 11 different short researcher-administered tasks to measure young children’s social cognition. Tasks were ordered from those that the youngest children should be able to complete, to those the oldest children should be able to complete, based on past literature. If children clearly failed three tasks in a row, the session was ended early, but participant data was still retained for analyses, and scores were based on the tasks completed to that point. For instance, if a child passed the 1^st^ and 3^rd^, task, and then failed tasks 4–6, the experiment ended, and their total score would be 2. This was because our study included children from a wide age range, from 3 to 47 months. Therefore, we did not expect, e.g., children under 1 year, to perform well on later tasks (e.g., answering verbal questions), and used this rule to end the session early when children clearly could not proceed, so as to avoid any stress for participants. All tasks were video recorded and coded from video.

##### Task 1: Joint attention (Carpenter, Nagell, et al., 1998) (previously all children passed at 9 months)

This task examined whether children alternate gaze between a person and object. The experimenter played with a toy watering can in silence while the experimenter alternated her gaze between the child and the object. The episode lasted around 15 s or until the child looked from the object to the experimenter’s face and back to the same object. Children scored one point if they looked from the object to the experimenter’s face and back to the same object, thus coordinating attention to both the adult and the object.

##### Task 2: Own intention (Carpenter, Nagell, et al., 1998) (previously all children passed at 9 months)

This task was used to measure whether children intentionally remove an obstacle to reach a target object. A toy goat was placed on the table in front of the child. A transparent plastic box was positioned upside down over the toy such that the child could see the toy but could not obtain it without moving the box. Then the experimenter said, “Can you get the toy?” and waited up to 10 s for a response. If the child did not succeed, the experimenter repeated the verbal prompt one more time. Children scored one point if they removed the obstacle.

##### Task 3: Pointing (Camaioni et al., 2004) (previously children passed at 11 months)

This task was used to determine whether children would point to share attention with another person. The experimenter made a toy bird fly around for 10 s. The experimenter hid the bird behind her back, so that the child could not see it. The experimenter said, “What happened?” and waited up to 5 s for a response. If there was no response, the experimenter repeated the question and waited for up to another 5 s. Children scored one point if they pointed to the object or gave a verbal cue asking for it.

##### Task 4: Point following (Carpenter, Nagell, et al., 1998) (previously children passed at 11 months)

This task measures whether children look where an adult points. The experimenter gave the child a toy carrot to play with. Then the experimenter put two different cubes in two separate locations on the table. The experimenter pointed to one of the cubes with her right hand while alternating her gaze between the child’s eyes and the target cube. The experimenter’s pointing continued either until the child fixated on the shape or once around 10 s had passed. Children scored one point if they first looked to the toy that the experimenter pointed to.

##### Task 5: Gaze following (Carpenter, Nagell, et al., 1998) (previously children passed at 11 months)

This task measures whether children look where an adult looks. The experimenter gave the child a blue dog toy to play with. Then the experimenter put two blocks in two separate locations on the table. The experimenter turned her head between the child and one of the blocks up to ten times. The experimenter’s head turns continued either until the child fixated on the target block or until the ten head turns were complete. Children scored one point if they looked to the block that the experimenter gazed at first.

##### Task 6: Mimicry (Carpenter, Nagell, et al., 1998) (previously children passed at 12 months)

This task measures whether children copy arbitrary actions. The experimenter patted the plastic box with her hand several times and smiled. The experimenter oriented the box toward the child and said, “Can you do that?” and gave the child around 5 s to copy. If there was no response, the experimenter repeated the action one more time and waited for another 5 s. Children scored one point if they reproduced the modelled action.

##### Task 7: Imitation, intentions, mistakes (Carpenter, Akhtar, et al., 1998; Carpenter, Nagell, et al., 1998) (previously children passed at 12 months)

This task measures whether children copy intentional actions, and avoid accidental actions. The experimenter flapped the top of a box and said “Whoops!” then pressed the purple button on the front of the box and said “There!” Then the experimenter waited for the flap to mechanically open showing a fish. The experimenter said, “Can you make it work?” and waited around 5 s for a response. If there was no response, the experimenter repeated the question and waited another 5 s. Children scored one point if they reproduced the intentional action, but not the accidental action. If children clearly were attempting to reproduce the intentional action but were unsuccessful owing to lack of strength/dexterity, they were given credit for reproducing that action.

##### Task 8: Desires (Repacholi & Gopnik, 1997) (previously children passed at 18 months)

This task measures whether children are aware of others’ desires. Two plates of food (broccoli and crackers) were presented and the experimenter said, “Try these!” and waited while the child tried. First, the experimenter tasted the child’s preferred food and acted disgusted and said, “Eww!” Second, the experimenter tasted the other food and said, “Yum!” and looked happy. The experimenter placed one hand, palm facing up, exactly between the two plates and said, “Can you give me some?” and waited for around 10 s. The experimenter repeated the question twice if necessary. Children scored one point if they offered their non-preferred food to the experimenter, showing they understood the experimenter’s desires, not just their own.

##### Task 9: Emotion (affective labeling task) (Denham, 1986) (previously children passed at 2 years)

This task measures whether children are aware of others’ emotions. The experimenter showed four pictures of children’s faces, with happy, sad, angry and afraid expressions. The experimenter asked, “How does this boy/girl feel?” and waited for around 10 s for a response. Children scored one point if they identified the correct emotions for at least three out of four pictures.

##### Task 10: Emotion (affective perspective-taking task, adapted from Denham, 1986, previously children passed at 2 years)

This task measures whether children understand that people can react emotionally differently than they themselves would in the same situation. Four pictures of children’s faces were placed in front of the child. The experimenter then used animal puppets to act through four scenarios – two in which the puppet’s emotional reactions were expected based on what occurred, and two in which the emotional reactions were unexpected. For example, in one scenario, the experimenter used a monkey puppet and said, “I have got an ice-cream, yay!” while showing a picture of an ice-cream. The experimenter asked the child, “How is the monkey feeling?” After each question, the experimenter waited around 10 s for a response and repeated the question if necessary. Children scored one point if they correctly identified how the puppets felt for at least three out of four scenarios.

##### Task 11: Beliefs (Sally- Anne task) (Baron-Cohen et al., 1985; Wellman et al., 2001) (previously children passed at 4.5 years)

This task measures whether children understand false beliefs. The experimenter introduced Sally and Anne, saying, “This is Sally and this is Anne.” The experimenter asked the child their names. “Who is she? Do you remember her name?” The experimenter then said, “Sally is putting the ball into her basket and then hides behind me. Anne is moving the ball into her own basket and leaves as well. When Sally returns, where will she look for the ball?” The experimenter waited for around 5 s for a response and repeated the question if there was no response. Children scored one point if they pointed to the previous location of the ball or said the previous location.

##### Coding

If children scored zero on three tasks in a row, coding stopped, and we summed the number of trials children passed up to this point for their final scores. This was to be consistent with our study’s stop rule, explained earlier. Scores were summed for an overall social cognition score. A second coder coded 17 (20%) of the videos. Agreement was very good, *Intra-class correlation* = 0.88.

### Results

See Table 3 for the descriptive statistics for children’s ages and ESCI scores, by year. Internal reliability for the 21 ESCI items was again excellent, *N* = 84, *KR20* = 0.93. All 11 researcher-administered social cognition tasks correlated with the total social cognition score (all *Spearman’s Rho r* > .41, *p* < .001, see Table 4 for all correlations). Internal reliability across the researcher-administered tasks was good, *KR20* = 0.80. Total scores on the researcher-administered tasks correlated strongly with the ESCI (*Pearson’s r* = .75, *p* < .001). A bootstrapped partial Pearson’s correlation (1000 samples), controlling for age in days (which was skewed), found a significant medium to large correlation between the total scores on the researcher-administered tasks and the ESCI (*r’* = .41, *p* < .001). There were no effects of, or interactions with, gender.

**Table 4:** Spearman’s Rho correlations between individual researcher-administered social cognition tasks, and total scores on the researcher-administered social cognition tasks

Task	*r*
Joint attention	.47*
Own intention	.54*
Pointing	.58*
Point following	.60*
Gaze following	.52*
Mimicry	.75*
Imitation, intentions, mistakes	.78*
Desires	.55*
Emotion: Affective labeling	.53*
Emotion: Affective perspective taking	.51*
Beliefs	.42*

*p < .05

### Discussion

Study 3 found that children’s scores on the ESCI correlated well with their scores on a battery of researcher-administered social cognition tasks, even when controlling for age. This suggests the ESCI has convergent validity. Study 4 sought to determine whether parents were consistent in their ESCI reporting. Therefore, a separate sample of parents completed the ESCI twice, 1 month apart to determine test–retest reliability.

## Study 4: 1-month test–retest reliability

### Method

#### Participants

For test–retest reliability, a power analysis found 29 children were needed for a two-tailed large correlation (*r* = 0.5) (Mayes et al., 1996; Tahiroglu et al., 2014), with *α* = 0.05, power = 0.8 (Faul et al., 2007). We aimed for 60 participants to account for attrition at Time 2. Sixty-three participants were recruited through the Cardiff University’s Centre for Human Developmental Science database. Forty-six participants repeated the survey 1 month later (28 male, 18 female, Time 1 *Mean* = 31 months, 12 days; *SD* = 11;21; *Range* = 7;22–47;25). However, we include demographic information for all 63 participants in Table 2 as their Time 1 data was used in Study 6. Participants were not paid.

#### Measures

##### ESCI

Parents completed the 21-item ESCI on their own computer through the website onlinesurveys.ac.uk. Four weeks later they were emailed to repeat the survey on the same website.

### Results

See Table 3 for the descriptive statistics for children’s ages and ESCI scores at Time 1, by year. On average, parents completed the ESCI 1 month and 5 days after previously completing it (*N* = 46; *SD* = 8.6 days; *Range* = 25 days to 2 months, 6 days). Total ESCI scores at Times 1 and 2 were negatively skewed, therefore we used bootstrapped Pearson correlations (1000 samples). ESCI scores at Times 1 and 2 were collinear (*r* = .932, *p* < .001). A bootstrapped partial correlation (1000 samples), controlling for age at Times 1 and 2, found a significant very large correlation between the ESCI at Times 1 and 2 (*r’* = .789, *p* < .001). There were no effects of, or interactions with, gender. We also examined whether there was a difference in ESCI scores at Times 1 (*M* = 16.46, *SD* = 4.27) and 2 (*M* = 16.83, *SD* = 4.55). There was no difference, bootstrapped paired-sample *t* test (2000 samples), *p* = .144.

### Discussion

Study 4 demonstrated that parents showed very good test–retest reliability over an interval of 1 month, even when controlling for age. The goal of Study 5 was to determine whether children’s scores remained stable over longer intervals: 6 and 12 months; and whether both parents gave similar scores to each other (inter-observer reliability). We also examined whether children’s scores increased significantly over 6- and 12-month time periods.

## Study 5: Longitudinal stability & inter-observer reliability

### Method

#### Participants

For longitudinal stability, a power analysis found 29 children were needed for a two-tailed large correlation (*r* = 0.5) (Mayes et al., 1996; Tahiroglu et al., 2014), with *α* = 0.05, power = 0.8 (Faul et al., 2007). Six-month longitudinal stability was run for a subsample of participants from Studies 1 and 2 (*N* = 140, 75 male, 65 female, Time 1 *Mean* = 21 months, 16 days; *SD* = 12 months, 26 days; *Range* = 23 days to 47 months, 9 days). Twelve-month longitudinal stability was run for another subsample of participants from Studies 1 and 2 (*N* = 39, 27 male, 12 female, Time 1 *Mean* = 13 months, 6 days; *SD* = 8 months, 24 days; *Range* = 1 month, 3 days to 30 months, 9 days). A power analysis found 13 children were needed for a two-tailed very large correlation (*r* = 0.7, since this is the minimum acceptable level for inter-observer reliability), with *α* = 0.05, power = 0.8 for inter-observer reliability between both parents (Faul et al., 2007). Inter-observer reliability was evaluated for a subsample of participants from Studies 1 and 2 (*N* = 36 pairs of parents, 18 male children, 18 female children, child’s age *Mean* = 17 months, 5 days; *SD* = 12 months, 6 days; *Range* = 1 month, 1 day – 44 months, 2 days for parent 1’s survey). Either up to £2 was donated to charity (e.g., UNICEF) for each survey that was repeated, or for when a second parent completed the survey; or participants received a £5 Amazon voucher (or equivalent in their country).

#### Measures

##### ESCI

For longitudinal stability, parents who originally completed the ESCI through www.babylovesscience.com were contacted by e-mail 6 and 12 months later to repeat the 21-item ESCI on the same website. Parents who originally completed the ESCI in the lab were contacted by e-mail 6 months later to repeat the ESCI through Qualtrics on their own computer. For inter-observer reliability, parents who completed the ESCI through www.babylovesscience.com were automatically told when they submitted their survey that we were looking for children’s other parents to complete the survey as well. The other parent completed the survey on www.babylovesscience.com.

### Results

#### Longitudinal stability – 6 Months

On average, parents in the 6-month longitudinal stability sample completed the ESCI 5 months and 22 days after previously completing it (*N* = 140; *SD* = 17 days; *Range* = 4 months, 25 days to 6 months, 26 days). Total ESCI scores at Times 1 and 2 were negatively skewed, therefore we use bootstrapped Pearson’s correlations (1000 samples). ESCI scores at Times 1 and 2 were nearly collinear (*r* = .898, *p* < .001). A bootstrapped partial correlation (1000 samples), controlling for age at Times 1 and 2, found a significant very large correlation between the ESCI at Times 1 and 2 (*r’* = .700, *p* < .001). There were no effects of, or interactions with, gender. We also examined whether there was a difference in ESCI scores at Times 1 and 2. Children’s scores were significantly higher at Time 2 (*M* = 15.33, *SD* = 5.21) than Time 1 (*M* = 12.36, *SD* = 6.44), using a bootstrapped paired-samples *t* test (2000 samples), *p* < .001.

#### Longitudinal stability – 12 Months

On average, parents in the 12-month longitudinal stability sample (some parents are the same as the 6-month sample, and some different) completed the ESCI 12 months and 22 days after previously completing it (*N* = 39; *SD* = 25 days; *Range* = 11 months, 18 days to 14 months, 10 days). Total ESCI scores at Time 2 were negatively skewed, therefore we use bootstrapped Pearson’s correlations (1000 samples). Total scores on the ESCI at Times 1 and 2 were very strongly correlated (Pearson’s *r* = .802, *p* < .001). A bootstrapped partial correlation (1000 samples), controlling for age at Times 1 and 2, found a significant large to very large correlation between the ESCI at Times 1 and 2 (*r’* = .641, *p* < .001). There were no effects of, or interactions with, gender. We also examined whether there was a difference in ESCI scores at Times 1 and 2. Children’s scores were significantly higher at Time 2 (*M* = 14.77, *SD* = 4.26) than Time 1 (*M* = 8.59, *SD* = 5.91), using a bootstrapped paired-samples *t* test (2000 samples), *p* < .001.

#### Inter-observer reliability

On average, where both parents completed the ESCI, they did so 1.83 days apart (*N* = 36 pairs of parents; *SD* = 5.65 days; *Range* = 0–24 days). Total scores on the ESCI for parents 1 and 2 were collinear (*Pearson’s r* = .960, *p* < .001). A partial correlation, controlling for ages when both parents completed the ESCI, found an almost collinear correlation between parents’ surveys (*r* = .871, *p* < .001). There were no effects of, or interactions with, child gender.

### Discussion

Children’s ESCI scores were relatively consistent after both 6 and 12 months, even when controlling for age, thus demonstrating developmental stability (Bornstein et al., 2017). Furthermore, children’s scores increased significantly over both 6 and 12 months, thus demonstrating developmental change (Bornstein et al., 2017). Additionally, when both parents completed the ESCI, their scores were almost collinear. Therefore, the ESCI shows good longitudinal stability and inter-observer reliability. Finally, in Study 6, we pool data across participants from Studies 1–4 to examine whether the ESCI is internally reliable within different demographic groups; to examine changes across ESCI items, and the ESCI as a whole, by age; and to examine whether there were any demographic differences.

## Study 6: Demographics

### Method

#### Participants

One of our goals in Study 6 was to examine demographic differences with small effect sizes. Therefore, we would need a total of 787 children for a two-tailed small correlation (*f* = 0.1) with *α* = 0.05, power = 0.8; for regression analyses including linear regression, ANOVA, and ANCOVA (Faul et al., 2007). Where we did not achieve these numbers due to parents not always choosing to report demographic variables, e.g., income, we could still look for demographic differences with 128 children for a two-tailed medium correlation (*f* = 0.25) with *α* = 0.05, power = 0.8. For the following analyses, we pooled data from all four samples from Studies 1, 2, and 3, and 4 (*N* = 1047, see Table 2).

#### Measures

##### ESCI

See Studies 1–4.

### Results

#### Reliability within different demographic groups

We first looked at whether the ESCI was internally reliable within different countries for which we had at least 16 participants. Internal reliability was excellent for participants in the United Kingdom (*N* = 686, *KR20* = 0.93), Australia (*N* = 133, *KR20* = 0.95), the United States (*N* = 103, *KR20* = 0.95), Canada (*N* = 25, *KR20* = 0.96), and Trinidad and Tobago (*N* = 16, *KR20* = 0.94).

We next looked at the ESCI’s internal reliability by parents’ education level. The ESCI’s internal reliability was excellent for participants who had a high school education (*N* = 118, *KR20* = 0.93), community college (*N* = 64, *KR20* = 0.94), an undergraduate degree (*N* = 387, *KR20* = 0.93), and a postgraduate degree (*N* = 460, *KR20* = 0.94).

We next looked at the ESCI’s internal reliability by parents’ ethnicity, where *N* was at least 16. The ESCI’s validity was excellent for parents who were black (including parents of mixed ethnicity, *N* = 26, *KR20* = 0.94), East Asian (including parents of mixed ethnicity, *N* = 18, *KR20* = 0.88), South Asian (including parents of mixed ethnicity, *N* = 16, *KR20* = 0.89), and white (*N* = 901, *KR20* = 0.94).

We next looked at the ESCI’s internal reliability by children’s language. The ESCI’s validity was excellent for children who were monolingual (*N* = 798, *KR20* = 0.94), and multilingual (*N* = 194, *KR20* = 0.93).

### Age of emergence

To determine whether the ESCI could be used with the youngest and eldest age groups, we examined internal reliability for each 2-month interval. We chose 2-month intervals because that ensured we had at least *N* = 16 participants per group. Within each age group, total ESCI score outliers were cut, where outliers were more than 3 standard deviations from the mean. From 4–5 up through 38–39 month groupings, internal reliability was acceptable, *KR20* = 0.65 – 0.85 (see Table 5). However, internal reliability was not acceptable under 4 months or over 40 months, *KR20* = – 0.62 to .57.

**Table 5:** *KR20* scores for each 2-month age grouping, from 0–47 months

Age (months)	*N*	Items	2-month *KR20*
0–1	17	5	– 0.62
2–3	29	8	0.57
4–5	40	15	0.69
6–7	34	15	0.81
8–9	38	18	0.76
10–11	48	17	0.82
12–13	58	21	0.85
14–15	60	19	0.67
16–17	69	19	0.69
18–19	51	21	0.71
20–21	44	18	0.67
22–23	51	16	0.73
24–25	52	21	0.73
26–27	40	20	0.65
28–29	52	19	0.76
30–31	49	18	0.78
32–33	46	17	0.65
34–35	29	13	0.69
36–37	36	17	0.68
38–39	35	15	0.78
40–41	34	12	0.54
42–43	32	12	0.27
44–45	41	16	0.49
46–47	29	13	0.45

*Note.* Items indicates the number of items showing variability for each age group.

In order to get an idea of when each ESCI item emerges, Appendix A shows the proportion of children reported to pass each item in each 2-month age group. To give a clearer picture, we also collapsed all data from all studies (excluding outliers, *N* = 1014) and ran stepwise binary logistic regressions with each ESCI item as the dependent variable, and age in 2-month intervals, age squared, and age cubed, as the independent variables. We then plotted the predicted proportion of children passing each item, by age (see Appendix A). Table 2 summarizes the age at which 25, 50, and 75% of children are predicted to pass each item according to these models.

**Fig. 2:**
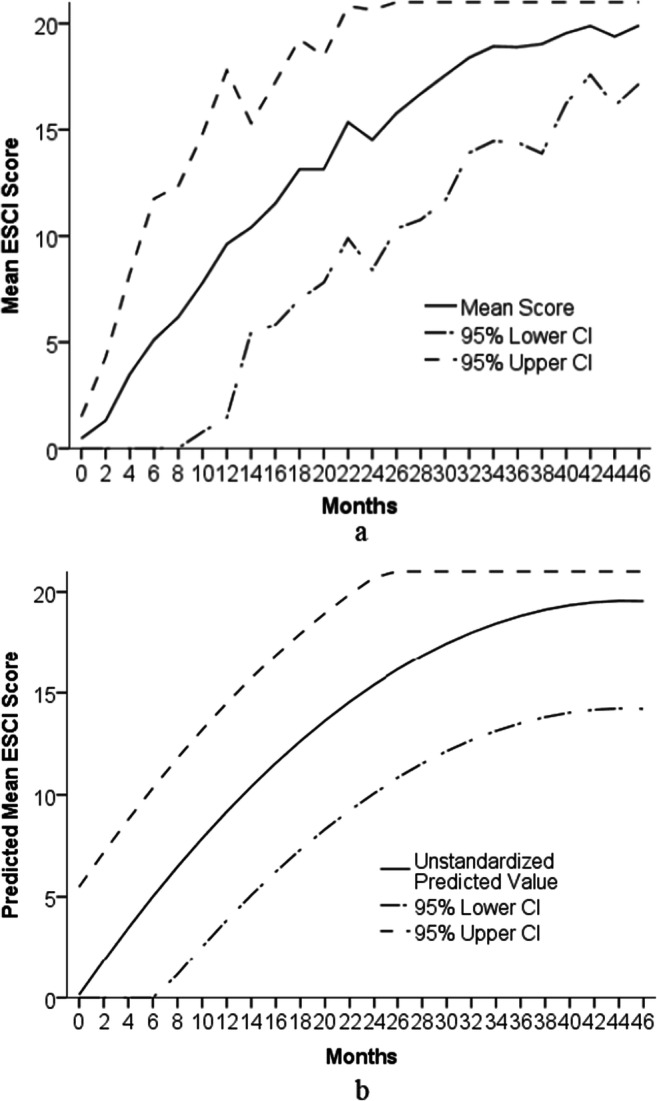
Mean ESCI scores with 95% individual confidence intervals (CIs) for each 2-month age group (Fig. 2a) and predicted mean ESCI scores, with predicted 95% individual CIs. *Note* See Table 5 for *N* for each age group. We changed CIs below 0 to 0, and above 21 to 21 to remain within the realm of possible scores

To give us a picture of expected socio-cognitive development by age, Fig. 2 shows the mean total ESCI scores for each 2-month age group (excluding outliers), and we also plotted 95% individual confidence intervals (CI; calculated as 2 standard deviations above and below each mean). Where these scores were impossible (under 0, over 21) we plotted the minimum (0) and maximum (21) scores instead. For each age group, we are 95% confident that the mean score is above the lower confidence interval, and may give an idea of when children would show particular advances, delays, or differences, in socio-cognitive development. For example, in Fig. 2a, by 26 months, we are 95% confident that the mean score is over 10 on the ESCI (based on the lower CI being 2 standard deviations below the mean), suggesting that, the ESCI may be useful for identifying children with socio-cognitive developmental differences, where children of this age score much lower than 10.

To get a clearer picture of how the ESCI changes with age, we also ran a bootstrapped linear regression with 2000 samples (as age was positively skewed, and ESCI scores were negatively skewed) on the total ESCI score as the dependent variable, entering age in 2-month intervals, then age squared, and then age cubed, as the independent variables for all children. The initial model found that while there were significant effects of age and age squared, there was no significant effects of age cubed, *B* = 0.000, *p* = .510. Therefore, we re-ran the analysis without age cubed. The model, *N* = 1014, *F*(2, 1011) = 1996.60, *p* < .001, found age in months, *B* = 0.869, *p* < .001, and age in months squared, *B* = – .010, *p* < .001, both predicted the ESCI. We then plotted the predicted ESCI scores of children, by age, as well as 95% individual confidence intervals (see Fig. 2b). We plotted this figure to give a clearer idea of what scores we might expect children to achieve at different ages. For example, based on this prediction, we would expect children to score at least 1 from 8 months, in line with the raw data.

### Demographic differences

We pooled data across all three samples from 4–39 months excluding age outliers (*N* = up to 832) to determine which factors correlated with ESCI scores with a small effect size. Since child age (2-month intervals) and the square of child age were strong correlates of the ESCI, we always included these variables in the models. We ran bootstrapped ANCOVAs (2000 samples) with ESCI scores as the dependent variables; child age and the square of child as covariates; and either child gender, parent gender, language (mono or multilingual), siblings, or country (where *N* at least 16 per country), as the independent variable. The overall model including siblings was significant, *N* = 810, *F*(3, 806) = 655.43, *p* < .001, *η*_*p*_^*2*^ = .709, such that there were significant effects of age, *F*(1, 806) = 316.64, *p* < .001, *η*_*p*_^*2*^ = .282, age-squared, *F*(1, 806) = 69.31, *p* < .001, *η*_*p*_^*2*^ = .079, and siblings, *F*(1, 806) = 10.80, *p* = .001, *η*_*p*_^*2*^ = .013. Children with siblings had significantly higher ESCI scores (unstandardized score controlling for age and age squared: *N* = 337; *M* = 13.87, *SD* = 4.53) than children without (*N* = 473, *M* = 12.22, *SD* = 4.39). The overall model for country was also significant, *N* = 896, *F*(5, 890) = 673.60, *p* < .001, *η*_*p*_^*2*^ = .791, such that there were significant effects of age, *F*(1, 890) = 861.66, *p* < .001, *η*_*p*_^*2*^ = .492, age-squared, *F*(1, 890) = 262.32, *p* < .001, *η*_*p*_^*2*^ = .228, and country, *F*(3, 890) = 7.32, *p* < .001, *η*_*p*_^*2*^ = .024. Post hoc Bonferroni pairwise comparisons found that Australian children (unstandardized score controlling for age and age squared: *N* = 106, *M* = 10.23, *SD* = 4.99) scored significantly lower than American children (*p* = .009, *M* = 12.91, *SD* = 5.74), British children (*p* < .001, *N* = 666, *M* = 14.22, *SD* = 4.92), and Canadian children (*p* = .025, *N* = 24, *M* = 12.06, *SD* = 5.88). No other differences were found between countries. None of the other demographic variables were significant (child gender, *N* = 831, *p* = .225; parent gender, *N* = 832, *p* = .395; language, *N* = 801, *p* = .759). We also ran bootstrapped linear regression models (2000 samples) with the ESCI as the dependent variable; child age and the square of child age as independent variables in step 1; and either parent age, parent education, or childcare hours, as the independent variable in step 2. Since we did not ask about childcare hours with our initial participants, we could only look for a medium effect size as *N* = 474. None of the demographic variables were significant predictors of the ESCI (parent age, *N* = 812, ΔF(1, 809) = 1.72, *p* = .190; parent education, *N* = 815, ΔF(1, 812) = 0.53, *p* = .467; childcare hours, *N* = 474, ΔF(1, 471) = 3.17, *p* = .076). Since we could only examine household income within countries, we could only look for a medium effect size of income within the United Kingdom due to sample size, *N* = 377. We ran bootstrapped linear regression models with the ESCI as the dependent variable; child age and the square of child age as independent variables in step 1; and household income in step 2. Household income significantly improved the model (see Table 6), such that the higher the household income, the higher the child’s ESCI score, controlling for age and age squared.

**Table 6:** Bootstrapped linear regression model fitting age (in 2-month intervals) and age squared in step 1; and household income in step 2; to the ESCI, for participants from the United Kingdom only

Model	*F Model*	*p*	*R*^*2*^		*B*	*p*
1	(2, 375) = 553.64	< .001	.747			
				Child age (2-month intervals)	.970	< .001
				Child age squared	– .012	< .001
2	Change:					
	(1, 374) = 5.28	.022	.004			
				Child age (2-month intervals)	.983	< .001
				Child age squared	– .012	< .001
				Household income	7.083E^–6^	.045

*Note. B* is a non-standardized coefficient.

### Discussion

The ESCI had internal reliability across a variety of demographic groups including country, parents’ education, parents’ ethnicity, and children’s language (mono- or multi-lingual). Looking at smaller age intervals, the ESCI showed internal reliability from 4–39 months. There were some demographic differences. As expected, children scored higher on the ESCI as they aged. Australian children were reported to have significantly lower ESCI scores than American, British, and Canadian children. Furthermore, children who had siblings had higher scores than children who did not, and ESCI scores increased with household income in the United Kingdom.

## General discussion

Our aims were to construct and evaluate a short parent-report measure of social cognition appropriate for children from birth to 47 months. The current study found evidence of convergent validity and reliability of the 21-question ESCI as a measure of social cognition. The survey showed high internal reliability across separate groups of parents, and this extended to parents from different countries (Australia, United Kingdom, United States, Canada, Trinidad and Tobago), different educational backgrounds (from high school through postgraduate degrees), different parent ethnicities (Black, East Asian, South Asian, and White) and different age groups (4–39 months). The survey also showed good test–retest reliability at 1 month, and good longitudinal stability at both 6 and 12 months, as well as good inter-observer reliability between parents. Finally, the ESCI showed good convergent validity. The scores between the ESCI and objective measures of social cognition administered by a researcher demonstrated that parents are accurate reporters of socio-cognitive development, and conversely, that researcher-administered social cognition tasks are good at capturing everyday socio-cognitive development.

An important aspect of the ESCI is that it captures socio-cognitive development from 4 through 39 months. The fact that the ESCI repeatedly shows good internal reliability through high *KR20* scores suggests that social cognition can be captured by items covering a range of socio-cognitive concepts. This converges with previous research (Carpenter, Nagell, et al., 1998; Hutchins et al., 2012; Tahiroglu et al., 2014; Wellman & Liu, 2004), but extends it, as it shows that socio-cognitive development can be measured across a greater range of socio-cognitive skills, all the way from 4 through 39 months. While we found a two-factor structure to the ESCI, the first factor reflected social cognition more generally, while the second factor reflected age, which is not surprising, as the ESCI aimed to track development. The factors did not cluster into meaningful groups, such as understanding beliefs or intentions.

The ESCI adds to previous parent-report measures of social cognition as it can be used as early as 4 months while other surveys focussing on social cognition and pragmatics cannot be used until 18 months or later (CSUS, ToMI, the Language Use Inventory, LUI) (O ' Neill, 2007, 2009; Tahiroglu et al., 2014). The ESCI shows that parent report measures of social cognition are accurate in early infancy, with only 21 questions, taking less than 5 min. This survey therefore provides researchers with a new, efficient, and valuable tool to assess social cognition early on, while many aspects of social cognition, such as joint attention, pointing, and imitation, are still developing.

An advantage of a short parent-report measure is that it allows us to more easily collect data from a large number of participants in a short time frame. One study looking at 100 years of social psychology research found that the average effect size for social psychological effects is *r* = .21 (Richard et al., 2003). Therefore, to get the average effect size requires a sample of at least 175 participants, with *α* = 0.05, power = 0.8 (Faul et al., 2007). Given that this is just an average, around half of social psychological effects would require an even larger sample size to detect. While it is not clear whether developmental psychological effects have the same average effect size, our demographic analyses included samples of over 1000 participants giving us enough power to test for smaller effect sizes. This gives us greater confidence of both our significant and null findings. To individually test a comparable sample of participants on a battery of researcher-administered social cognition tasks would likely require a dedicated full-time research assistant and lab space for around 5 years in a mid-size city. Furthermore, to acquire data from five countries would require a collaboration across at least five labs. Using a parent-report measure is much more efficient.

Our results based on demographics found that, unsurprisingly, older children had higher social cognition scores, consistent with previous research (Hiller et al., 2014; Wellman et al., 2001; Wellman et al., 2011; Wellman & Liu, 2004). Furthermore, Australian parents reported lower levels of social cognition than American, British, and Canadian parents, once age was controlled for. This points to the possibility that children in Australia either have lower rates of social cognition more generally, or at least that parents perceive this. It is also possible that Australian parents interpreted the questions differently. This result counters that by Wellman et al. (2001) finding that Australian children passed theory of mind tasks at a significantly higher rate than American children. Children with siblings scored higher on the ESCI. This converges with research showing that children with siblings pass Theory of Mind tasks earlier than those without (Jenkins & Astington, 1996; Lewis et al., 1996; Perner et al., 1994; Ruffman et al., 1998). Finally, within the United Kingdom, the higher the household income, the higher children’s ESCI scores, converging with research suggesting SES is positively correlated with researcher-administered socio-cognitive measures (Cole & Mitchell, 1998), but diverging from past research finding that household income generally did not predict children’s researcher-administered social cognition measures (Pears & Moses, 2003; Weimer & Guajardo, 2005).

Despite our large sample sizes, we did not find significant results for other demographic variables including child gender, childcare hours, parent age, parent gender, parent education, or language (mono versus multilingualism, when we controlled for child age and child age squared). It is important to note that participants from Samples 1 and 2 were self-selected through responding to Facebook adverts and posts, an article on a parenting website, and word of mouth. Therefore, they may not be representative of their country, education level, or other demographic factors. However, a benefit of online recruitment is that parents did not need to live in a university town to participate, nor did they need several hours during typical working hours to take time to participate in a study, suggesting these samples are more likely broader and more representative than standard lab samples. Indeed, the participants we recruited in our online studies were more ethnically and educationally diverse on average than those in our researcher-administered study. A post hoc analysis found that while 14% of children recruited through www.babylovesscience.com were from Black and Minority Ethnic (BAME) backgrounds, in line with the 2011 United Kingdom Census results for ethnicity (also 14% from BAME backgrounds) (Office for National Statistics et al., 2016), only 6% of children from our lab databases were from BAME backgrounds. Similarly, while 18% of parents recruited through www.babylovesscience.com did not have a university degree, slightly fewer parents recruited through our lab databases (15%) did not have a university degree. However, it should be noted that only around 27% of United Kingdom residents had a university qualification in 2011 (Office for National Statistics et al., 2016), suggesting our participants’ education levels were non-representative overall.

The ESCI provides a useful tool in a variety of research situations. Future research can use the ESCI to control for individual differences in social cognition for researcher-administered tasks or survey-based studies where social cognition might be relevant. Additionally, the ESCI can be used to efficiently examine how social cognition might correlate to, and predict, other areas of development, such as language, play, or social behavior. Finally, our age predictions, based on over 1000 participants, may be useful to educators, parents, and practitioners for understanding patterns of atypical social-cognitive development in children with neurodevelopmental conditions or additional support needs. For instance, at 26 months, we are 95% confident that the mean is at least 1 on the ESCI, suggesting children scoring 0 may need attention in terms of a referral for diagnosis, consideration of developmental delay or difference, or additional parental or educational input. Indeed, our raw data found that all 52 children in the 26-27 month age range scored at least 8. While larger sample sizes and replication would be needed to use the ESCI in this way, it shows good potential for this purpose.

## Limitations

There are several limitations with the ESCI. First, it does not work very well for children under 4 months, or over 39 months. This is likely due to the low number of items with variability in each of these age ranges (see Table 5). A related limitation is that the ESCI does not cover all socio-cognitive skills in the age range. For instance, our items did not including emotion mimicry (Isomura & Nakano, 2016) or humor (O ' Neill, 2007; Tahiroglu et al., 2014). Therefore, the ESCI really captures a subset of socio-cognitive skills, rather than social cognition as a whole in this age range. This was in part intentional, as we wanted the survey to be short and efficient. However, future research could examine whether the ESCI could be broadened by including a wider array of socio-cognitive skills so that social cognition is better captured overall, particularly focussing on items that show variability under 4 months and over 39 months.

A second limitation of the ESCI is that some of the items may be worded in a manner that is confusing for parents, and asking “Yes/No” questions may lessen the sensitivity of the ESCI. While we showed good internal reliability across countries and parent education level, it is still possible that some items, such as those involving the Latin term “e.g.,” might be confusing for parents. Furthermore, since Australian parents scored their children lower than children in Canada, the United Kingdom, and the United States, it is possible that Australian parents interpreted the questions differently. Future research should interview parents to determine whether items make sense to them (DeVellis, 2017). Finally, in future, we could test whether using a Likert scale may better capture individual differences and reliability for the ESCI. This may be particularly useful for including children over 39 months, who were at ceiling on several items.

A final limitation is related to sampling. First of all, as we could not include children who refused to participate in the researcher-administered tasks in Study 3 (*N* = 26), we may only have included a certain type of child, e.g., those who were better at socio-cognitive tasks overall, or those who were e.g., more outgoing. This could have affected the results of Study 3 as the sample may have been self-selected by individual differences. Similarly, as some developmental differences, such as autism spectrum disorder (ASD), are generally not diagnosed early on (Landa & Garrett-Mayer, 2006), our sample may have included children with developmental differences. If this is the case, this could have changed our factor structures, reliabilities, or demographic results. However, a benefit of the ESCI is that it could potentially in future be used to look at differences between children with typical development and developmental differences, such as ASD, or could be examined in conjunction with other individual difference measures, such as temperament (Putnam et al., 2006). Additionally, we did not consider the type of parents who completed the ESCI. For instance, Parent et al. (2010) discuss how parents with depression may be less mindful in their parenting. Therefore, if parents have depression, their answers on the ESCI may not be as accurate. Although our high inter-observer reliability between parents indirectly suggests both primary and secondary caregivers are good at reporting. Future research should examine how the ESCI works with different populations of caregivers.

## Conclusions

The ESCI is an efficient survey capturing a subset of socio-cognitive skills. It is reliable for use in children from 4–39 months, and may be useful with children as young as birth, or up to 47 months, when used within a wide age range. The ESCI shows good internal reliability, a consistent factor structure, and good test–retest reliability, inter-observer reliability, and longitudinal stability at 6 and 12 months. The ESCI may be useful in future research to efficiently examine how socio-cognitive development may link to other areas of development, or to act as a control measure in socio-cognitive experiments. Furthermore, with further development, it may be a useful tool to identify children with different developmental profiles than children with typical development.

### Supplementary Information

ESM 1 (CSV 34 kb)

ESM 2 (CSV 71 kb)

ESM 3 (CSV 12 kb)

ESM 4 (CSV 8 kb)

ESM 5 (CSV 4 kb)

ESM 6 (CSV 13 kb)

ESM 7 (CSV 3 kb)

ESM 8 (CSV 3 kb)

## **Appendix A** Age curves for each item

Item 1: Does your child follow where you look in order to look at the same thing as you?

Item 2: Is your child aware of other people’s motives? E.g., that they might give someone a gift in order to make them happy.

Item 3: Is your child aware of their own desires? E.g., prefer chocolate over broccoli.

Item 4: Is your child aware that other people may know the same information they do? E.g., they know where a certain book is kept, and they know their dad knows where that book is kept too.

Item 5: Is your child aware of other people ' s perspectives? E.g., could they tell sometimes they can see something, but someone else can’t, because it’s not in their line of sight.

Item 6: Is your child aware of his/her own mistakes? E.g., if s/he drops something by accident.

Item 7: Does your child perform actions intentionally? E.g., stack blocks on purpose, instead of by trial and error.

Item 8: Does your child follow where you point to look at the same things as you?

Item 9: Does your child look back and forth between you and an object, instead of only looking at you or only at an object?

Item 10: Does your child understand that sometimes things aren’t as they appear? E.g., something that looks hard might feel soft.

Item 11: Does your child copy others in order to achieve the same goal? E.g., copying pressing a button to make a song play on a toytpins.

Item 12: Is your child aware that sometimes other people don’t have the same beliefs as them? E.g., your child might think dogs are the best animal, but they understand that their sister thinks cats are the best animal.

Item 13: Is your child aware of their own emotions? E.g., happy, sad, angry, etc.

Item 14: Does your child point to get something from you? E.g., to get a toy that is out of reach.

Item 15: Does your child understand that sometimes other people have different desires to themselves? E.g., other people might like broccoli, even if they don’t.

Item 16: Does your child point to share information with you? E.g., point to show you a dog in the park.

Item 17: Is your child aware of other people’s emotions? E.g., happy, sad, angry, etc.

Item 18: Is your child aware that other people may have the same beliefs as them? E.g., that dogs are the best animals.

Item 19: Is your child aware that sometimes other people don’t know the same information they do? E.g., child might know where a toy is, but dad might not.

Item 20: Does your child understand what it means for others to make mistakes? E.g., that they dropped a plate by accident.

Item 21: Does your child perform actions with specific goals in mind? E.g., stacking blocks specifically to make a house.

## References

[CR1] Bakeman R, Adamson LB (1984). Coordinating attention to people and objects in mother–infant and peer–infant interaction. Child Development.

[CR2] Barna J, Legerstee M (2005). Nine-and twelve-month-old infants relate emotions to people ' s actions. Cognition & Emotion.

[CR3] Baron-Cohen S, Leslie AM, Frith U (1985). Does the Autistic child have a Theory of Mind. Cognition.

[CR4] Behne T, Liszkowski U, Carpenter M, Tomasello M (2012). Twelve-month-olds ' comprehension and production of pointing. British Journal of Developmental Psychology.

[CR5] Bornstein MH, Putnick DL, Esposito G (2017). Continuity and stability in development. Child Development Perspectives.

[CR6] Brooks R, Meltzoff AN (2005). The development of gaze following and its relation to language. Developmental Science.

[CR7] Camaioni L, Perucchini P, Bellagamba F, Colonnesi C (2004). The role of declarative pointing in developing a Theory of Mind. Infancy.

[CR8] Carpenter M, Akhtar N, Tomasello M (1998). Fourteen through 18-month-old infants differentially imitate intentional and accidental actions. Infant Behavior & Development.

[CR9] Carpenter M, Call J, Tomasello M (2005). Twelve-and 18-month-olds copy actions in terms of goals. Developmental Science.

[CR10] Carpenter M, Nagell K, Tomasello M (1998). Social cognition, joint attention, and communicative competence from 9 to 15 months of age. Monographs of the Society for Research in Child Development.

[CR11] Choe DE, Lane JD, Grabell AS, Olson SL (2013). Developmental precursors of young school-age children ' s hostile attribution bias. Developmental Psychology.

[CR12] Cole K, Mitchell P (1998). Family background in relation to deceptive ability and understanding of the mind. Social Development.

[CR13] Csibra, G., & Gergely, G. (2006). Social learning and social cognition: The case for pedagogy. Processes of change in brain and cognitive development. Attention and performance XXI, 21, 249-274

[CR14] Curenton SM (2011). Understanding the landscapes of stories: The association between preschoolers’ narrative comprehension and production skills and cognitive abilities. Early Child Development and Care.

[CR15] Denham SA (1986). Social cognition, prosocial behavior, and emotion in preschoolers: Contextual validation. Child Development.

[CR16] Denham SA, Caverly S, Schmidt M, Blair K, DeMulder E, Caal S, Hamada H, Mason T (2002). Preschool understanding of emotions: Contributions to classroom anger and aggression. Journal of Child Psychology and Psychiatry.

[CR17] DeVellis, R. F. (2017). *Scale development: Theory and applications (4th ed.)*. Sage.

[CR18] Farroni T, Johnson MH, Menon E, Zulian L, Faraguna D, Csibra G (2005). Newborns ' preference for face-relevant stimuli: Effects of contrast polarity. Proceedings of the National Academy of Sciences.

[CR19] Faul F, Erdfelder E, Lang A-G, Buchner A (2007). G*Power 3: A flexible statistical power analysis program for the social, behavioral, and biomedical sciences. Behavior Research Methods.

[CR20] Flavell JH, Flavell ER, Green FL (1983). Development of the appearance–reality distinction. Cognitive Psychology.

[CR21] Flavell JH, Flavell ER, Green FL, Moses LJ (1990). Young children ' s understanding of fact beliefs versus value beliefs. Child Development.

[CR22] Frith CD (2008). Social cognition. Philosophical Transactions of the Royal Society of London B: Biological Sciences.

[CR23] Gattis, M. (2018). Social cognition. In M. Bornstein (Ed.), The *SAGE encyclopedia of lifespan human development* (pp. 2033–2034 ). Sage Publications Inc. 10.4135/9781506307633.n756

[CR24] Gauvain M, Greene JK (1994). What do young children know about objects?. Cognitive Development.

[CR25] Gergely G, Bekkering H, Kiraly I (2002). Rational imitation in preverbal infants. Nature.

[CR26] Gopnik A, Slaughter V (1991). Young children ' s understanding of changes in their mental states. Child Development.

[CR27] Hilbrink EE, Sakkalou E, Ellis-Davies K, Fowler NC, Gattis M (2013). Selective and faithful imitation at 12 and 15 months. Developmental Science.

[CR28] Hiller RM, Weber N, Young RL (2014). The validity and scalability of the Theory of Mind Scale with toddlers and preschoolers. Psychological Assessment.

[CR29] Hutchins TL, Prelock PA, Bonazinga L (2012). Psychometric evaluation of the Theory of Mind Inventory (ToMI): A study of typically developing children and children with autism spectrum disorder. Journal of Autism and Developmental Disorders.

[CR30] Isomura T, Nakano T (2016). Automatic facial mimicry in response to dynamic emotional stimuli in five-month-old infants. Proceedings of the Royal Society B: Biological Sciences.

[CR31] Jenkins JM, Astington JW (1996). Cognitive factors and family structure associated with theory of mind development in young children. Developmental Psychology.

[CR32] Johnson MH, Dziurawiec S, Ellis H, Morton J (1991). Newborns ' preferential tracking of face-like stimuli and its subsequent decline. Cognition.

[CR33] Jones SS (2007). Imitation in infancy: The development of mimicry. Psychological Science.

[CR34] Kovács AM, Tauzin T, Téglás E, Gergely G, Csibra G (2014). Pointing as epistemic request: 12-month-olds point to receive new information. Infancy.

[CR35] Laakso M, Poikkeus A, Katajamaki J, Lyytinen P (1999). Early intentional communication as a predictor of language development in young toddlers. First Language.

[CR36] Landa R, Garrett-Mayer E (2006). Development in infants with autism spectrum disorders: A prospective study. Journal of Child Psychology and Psychiatry.

[CR37] Lewis C, Freeman NH, Kyriakidou C, Maridaki Kassotaki K, Berridge DM (1996). Social influences on false belief access: Specific sibling influences or general apprenticeship?. Child Development.

[CR38] Libertus K, Needham A (2014). Face preference in infancy and its relation to motor activity. International Journal of Behavioral Development.

[CR39] Liszkowski U (2005). Human twelve-month-olds point cooperatively to share interest with and helpfully provide information for a communicative partner. Gesture.

[CR40] Liszkowski U, Carpenter M, Henning A, Striano T, Tomasello M (2004). Twelve-month-olds point to share attention and interest. Developmental Science.

[CR41] Liszkowski U, Carpenter M, Striano T, Tomasello M (2006). 12-and 18-month-olds point to provide information for others. Journal of Cognition and Development.

[CR42] Liszkowski U, Carpenter M, Tomasello M (2007). Pointing out new news, old news, and absent referents at 12 months of age. Developmental Science.

[CR43] Mayes LC, Klin A, Tercyak KP, Cicchetti DV, Cohen DJ (1996). Test–retest reliability for false-belief tasks. Journal of Child Psychology and Psychiatry.

[CR44] McGillion M, Herbert JS, Pine J, Vihman M, DePaolis R, Keren-Portnoy T, Matthews D (2017). What paves the way to conventional language? The predictive value of babble, pointing, and socioeconomic status. Child Development.

[CR45] Meltzoff, A. N. (2007). ‘Like me’: a foundation for social cognition. Developmental science, 10(1), 126-134. 10.1111/j.1467-7687.2007.00574.x 10.1111/j.1467-7687.2007.00574.x PMC1852489 17181710

[CR46] Miller SE, Marcovitch S (2015). Examining executive function in the second year of life: Coherence, stability, and relations to joint attention and language. Developmental Psychology.

[CR47] Moberg SA, Ng R, Johnson DE, Kroupina MG (2017). Impact of joint attention on social-communication skills in internationally adopted children. Infant Mental Health Journal.

[CR48] Moll H, Tomasello M (2006). Level 1 perspective-taking at 24 months of age. British Journal of Developmental Psychology.

[CR49] Morales M, Mundy P, Delgado CE, Yale M, Messinger D, Neal R, Schwartz HK (2000). Responding to joint attention across the 6- through 24-month age period and early language acquisition. Journal of Applied Developmental Psychology.

[CR50] O ' Neill DK (2007). The Language Use Inventory for young children: A parent-report measure of pragmatic language development for 18- to 47-month-old children. Journal of Speech, Language, and Hearing Research.

[CR51] O ' Neill, D. K. (2009). *Language Use Inventory: An assessment of young children’s pragmatic language development for 18-to 47-month-old children [Manual]*. Knowledge in Development. [Record #387 is using a reference type undefined in this output style.]

[CR52] Office for National Statistics, National Records of Scotland, & Northern Ireland Statistics and Research Agency. (2016). 2011 Census aggregate data. 10.5257/census/aggregate-2011-1

[CR53] Onishi KH, Baillargeon R (2005). Do 15-month-old infants understand false beliefs?. Science.

[CR54] Parent J, Garai E, Forehand R, Roland E, Potts J, Haker K, Champion JE, Compas BE (2010). Parent mindfulness and child outcome: The roles of parent depressive symptoms and parenting. Mindfulness.

[CR55] Pears KC, Moses LJ (2003). Demographics, parenting, and theory of mind in preschool children. Social Development.

[CR56] Pedhazur, E. J., & Schmelkin, L. P. (1991). *Measurement, design, and analysis: An integrated analysis*. Erlaum.

[CR57] Perner J, Ruffman T, Leekam SR (1994). Theory of mind is contagious: You catch it from your sibs. Child Development.

[CR58] Perra O, Gattis M (2010). The control of social attention from 1 to 4 months. British Journal of Developmental Psychology.

[CR59] Perra O, Gattis M (2012). Attention engagement in early infancy. Infant Behavior & Development.

[CR60] Putnam SP, Gartstein MA, Rothbart MK (2006). Measurement of fine-grained aspects of toddler temperament: The Early Childhood Behavior Questionnaire. Infant Behavior and Development.

[CR61] Repacholi BM, Gopnik A (1997). Early reasoning about desires: Evidence from 14-and 18-month-olds. Developmental Psychology.

[CR62] Revelle, W. (2014). *The ’psych’ package* In http://cran.r-project.org/web/packages/psych/index.html

[CR63] Richard FD, Bond CF, Stokes-Zoota JJ (2003). One hundred years of social psychology quantitatively described. Review of General Psychology.

[CR64] Ruffman T, Perner J, Naito M, Parkin L, Clements WA (1998). Older (but not younger) siblings facilitate false belief understanding. Developmental Psychology.

[CR65] Sakkalou E, Ellis-Davies K, Fowler NC, Hilbrink EE, Gattis M (2013). Infants show stability of goal-directed imitation. Journal of Experimental Child Psychology.

[CR66] Sakkalou E, Gattis M (2012). Infants infer intentions from prosody. Cognitive Development.

[CR67] Selcuk B, Brink KA, Ekerim M, Wellman HM (2018). Sequence of Theory-of-Mind acquisition in Turkish children from diverse social backgrounds. Infant and Child Development.

[CR68] Shahaeian A, Peterson CC, Slaughter V, Wellman HM (2011). Culture and the sequence of steps in Theory of Mind development. Developmental Psychology.

[CR69] Slaughter V, Dennis MJ, Pritchard M (2002). Theory of Mind and peer acceptance in preschool children. British Journal of Developmental Psychology.

[CR70] Southgate V, Chevallier C, Csibra G (2009). Sensitivity to communicative relevance tells young children what to imitate. Developmental Science.

[CR71] Southgate V, Chevallier C, Csibra G (2010). Seventeen-month-olds appeal to false beliefs to interpret others referential communication. Developmental Science.

[CR72] Southgate V, Senju A, Csibra G (2007). Action anticipation through attribution of false belief by 2-year-olds. Psychological Science.

[CR73] Starkweather, J. (2014). *Factor analysis with binary items: A quick review with examples* (Benchmarks RSS Matters, Issue.

[CR74] Tabachnick, B. G., & Fidell, L. S. (2007). *Using multivariate statistics*. Allyn & Bacon/Pearson Education.

[CR75] Tahiroglu D, Moses LJ, Carlson SM, Mahy CE, Olofson EL, Sabbagh MA (2014). The Children’s Social Understanding Scale: Construction and validation of a parent-report measure for assessing individual differences in children’s Theories of Mind. Developmental Psychology.

[CR76] Tomasello, M. (1995). Joint attention as social cognition. In C. Moore & P. Dunham (Eds.), *Joint attention: Its origins and role in development* (pp. 103–130). Erlbaum.

[CR77] Tsao FM, Liu HM, Kuhl PK (2004). Speech perception in infancy predicts language development in the second year of life: A longitudinal study. Child Development.

[CR78] Weimer AA, Guajardo NR (2005). False belief, emotion understanding, and social skills among Head Start and non-Head Start children. Early Education and Development.

[CR79] Wellman HM, Bartsch K (1988). Young children ' s reasoning about beliefs. Cognition.

[CR80] Wellman HM, Cross D, Watson J (2001). Meta-analysis of Theory-of-Mind development: The truth about false belief. Child Development.

[CR81] Wellman HM, Fang F, Liu D, Zhu L, Liu G (2006). Scaling of Theory-of-Mind understandings in Chinese children. Psychological Science.

[CR82] Wellman HM, Fang F, Peterson CC (2011). Sequential progressions in a Theory-of-Mind scale: Longitudinal perspectives. Child Development.

[CR83] Wellman HM, Liu D (2004). Scaling of Theory-of-Mind tasks. Child Development.

[CR84] Wellman HM, Woolley JD (1990). From simple desires to ordinary beliefs: The early development of everyday psychology. Cognition.

